# STING Signaling Promotes Apoptosis, Necrosis, and Cell Death: An Overview and Update

**DOI:** 10.1155/2018/1202797

**Published:** 2018-11-25

**Authors:** Song Liu, Wenxian Guan

**Affiliations:** Department of Gastrointestinal Surgery, Nanjing Drum Tower Hospital, The Affiliated Hospital of Nanjing University Medical School, Nanjing, China

## Abstract

STING is a newly identified intracellular sensor of foreign and endogenous DNA. STING has been recognized as an activator of immune responses by TBK1/IRF3 and NF-*κ*B pathways, and it is suggested to play critical roles in host defense, autoimmune diseases, and tumor immunity. Recent studies have revealed that the outcome of STING activation could vary between distinct cell types and scenarios. STING activation in certain cell types triggered cell death including apoptosis and necrosis. This effect could be critical for preventing unnecessary or excessive inflammatory events and maintaining host immune homeostasis. This review is dedicated to summarize recent evidences in the field of STING-mediated cell death and to demonstrate dual outcomes of STING signaling. Besides canonical immune responses represented by IFN and TNF productions, STING signaling can also induce cell death events in a variety of cell types. The double-faced characteristics of STING signaling requires further exploration and precious regulation before tailoring clinical strategies for associated diseases.

## 1. Introduction

STING (stimulator of interferon genes) has been recently recognized as a central part of the recognition of bacterial and viral DNA as well as endogenous DNA (e.g., mitochondrial DNA). A series of studies in the recent decade has demonstrated the critical role of STING signaling in host immune responses and therefore in autoimmune diseases and tumor immunity [1, 2].

In antigen-presenting cells (e.g., macrophages and dendritic cells), STING can cooperate with other molecules (e.g., cGAS) to recognize DNA for TBK1/IRF3 and NF-*κ*B pathway activation and subsequent IFN and TNF production, respectively [3, 4]. Also, STING is capable of directly sensing bacterial and viral messengers under certain conditions [3, 5, 6]. The cellular and molecular process of STING signaling in innate immunity has been well reviewed elsewhere [7–9]. The potential application of targeting the STING pathway for cancer immunotherapy has been reviewed as well [10]. The complex of STING agonist and nanoparticles has been tested in antitumor therapy. Following membrane rupture and oxidative stress of tumor cells by cytotoxic nanoparticles, STING activation can enhance antitumor immunity by increasing expansion of tumor-infiltrating antigen-presenting cells and CD8+ T cells [11].

Recent evidences are emerging to show that the outcome of STING activation could vary between distinct cell types (which will be discussed below). In contrast to significant immune responses by STING in cells of the innate immune system, STING activation in other cell types leads to contrary outcomes. This review will summarize recent evidences in the field of STING-mediated cell death (including apoptosis and necrosis) and discuss relevant clinical significance.

## 2. Overview of STING in Apoptosis

The interaction between STING signaling and apoptosis was firstly and independently reported by White et al. [12] and Rongvaux et al. [13] in 2014. They reported that STING is involved in Bak/Bax-mediated apoptosis. They discovered that Bak/Bax could induce mtDNA efflux that triggers the cGAS-STING pathway and subsequent IFN production. More importantly, they found that activated caspases are capable of suppressing this Bak/Bax-induced STING-mediated apoptosis and consequently preventing dying cells from triggering inflammatory events to maintain host immune homeostasis.

Recently, McArthur et al. have successfully identified the mechanism of mtDNA efflux during apoptosis [14]. They utilized live-cell lattice light-sheet microscopy to observe real-time mtDNA escape from the mitochondria during intrinsic apoptosis. They discovered that this process requires prodeath protein Bak/Bax to form macropores in the mitochondrial outer membrane that allows mtDNA efflux into the cytoplasm [15].

## 3. STING Promotes Apoptosis of T Lymphocytes

Larkin et al. provided the first evidence of STING activation in T cells [16]. They found that STING activation in T cells triggers canonical inflammatory IFN production and simultaneous T cell ER stress and death events. Consistently, Gulen et al. reported that cGAS-STING activation in T cells results in a different gene expression profile compared to that in dendritic cells and—more importantly—the induction of T cell apoptosis [17]. This finding could be especially helpful for developing new treatment strategies in the context of T cell-associated human tumors.

In addition to accelerating cell death, STING activation in T lymphocytes could also prevent cell proliferation. Cerboni et al. discovered that the antiproliferative capacity of STING requires STING relocalization to the Golgi apparatus and is dependent on a C-terminal subdomain that activates NF-*κ*B but is distinct from TBK1/IRF3 recruitment domains. They also confirmed that patients carrying constitutive mutations in the STING-encoding gene presented a reduced number of T memory cells and impaired T cell proliferation [18]. This clinical relevance could help to understand the pathogenesis of T cell-deficiency or T cell-dysfunction diseases.

## 4. STING Promotes Apoptosis of Myeloid Cells

The proapoptotic effect of STING is not limited to T cells. Sze et al. observed STING-mediated apoptosis in human myeloid lineage cells with clinical relevance [19]. They found that during human T cell leukemia virus type 1 (HTLV-1) infection, viral reverse transcription intermediates could cooperate with STING to promote the IRF3-Bax complex for subsequent apoptosis of human primary monocytes. This finding provides a mechanistic explanation of HTLV-1-associated myelopathies.

Gaidt et al. for the first time elucidated the mechanism by which STING regulates DNA-mediated inflammasome activation for cell death of human myeloid cells [20]. By detecting cytosolic DNA, STING traffics to the lysosome, where it induces membrane permeabilization and subsequent K^+^ efflux that activates NLRP3-associated inflammasomes. Lysosomal cell death is a programed form of cell death associated with the rupture of lysosomes and leakage of lysosomal content into the cytosol [21]. Although different from apoptosis, lysosomal cell death induced by STING can provide a target for ameliorating inflammation in myeloid cells.

Interestingly, T lymphocytes and myeloid cells can cooperate to induce tumor cell death upon the activation by the STING agonist. Weiss et al. demonstrated that the STING agonist causes breast tumor vasculature disruption and then recruits massive immune cells (including neutrophils, CD8+ T cells, and monocytes) into the tumor site. Subsequent tumor cell death relies on the cooperation between myeloid and T cell subsets [22]. This finding provides a new insight into the translational application of STING-targeted therapy in cancer.

## 5. STING Promotes Apoptosis of Other Cell Types

STING has been shown to be critical in tumor immunity largely due to its capacity for boosting host antitumor immune responses, such as IFN production. Recently, Tang et al. reported that STING agonists can directly eradicate malignant B cells by promoting B cell apoptosis that also contributes to host antitumor activity [23]. This finding offers a theoretic support of STING agonists being used as adjuvants for vaccinations and cancer therapy. Nevertheless, their data simultaneously showed the cytotoxic effect of STING on normal B cells. Therefore, clinical awareness is still required when using STING agonists in treating B cell-associated diseases (e.g., chronic lymphocytic leukemia and multiple myeloma).

Petrasek et al. identified the central role of STING-mediated apoptosis in the development of alcoholic liver disease [24]. They found that ethanol provokes ER stress as well as the ER adaptor—STING, which leads to downstream IRF3 phosphorylation that links to the Bak/Bax molecule and thereby contributing to hepatocyte apoptosis. Furthermore, Qiao et al. discovered that STING-associated apoptosis is also critical in nonalcoholic fatty liver disease (NAFLD) [25]. They reported that suppression of STING could attenuate hepatic inflammation and protect the hepatocyte from apoptosis in NAFLD.

## 6. STING Signaling and Necrosis

For the first time, Sarhan et al. reported the important role of STING in the induction of necrosis in 2017 [26]. They found that the natural low level of the host DNA activates cGAS-STING signaling, which is essential for constitutive IFN and the initiation of necrosis during the homeostasis state.

This conclusion was confirmed by Brault et al. recently [27]. By using a bone marrow-derived macrophage model, they identified that necrosis is a major outcome of STING signaling upon recognition of intracellular DNA. They further discovered a synergistic effect between IFN and TNF pathways for the induction of necrosis. This finding is interesting since both IFN and TNF are downstream of STING signaling, which strongly implies the critical role of STING in necrosis.

## 7. Conclusion

**Figure 1:**
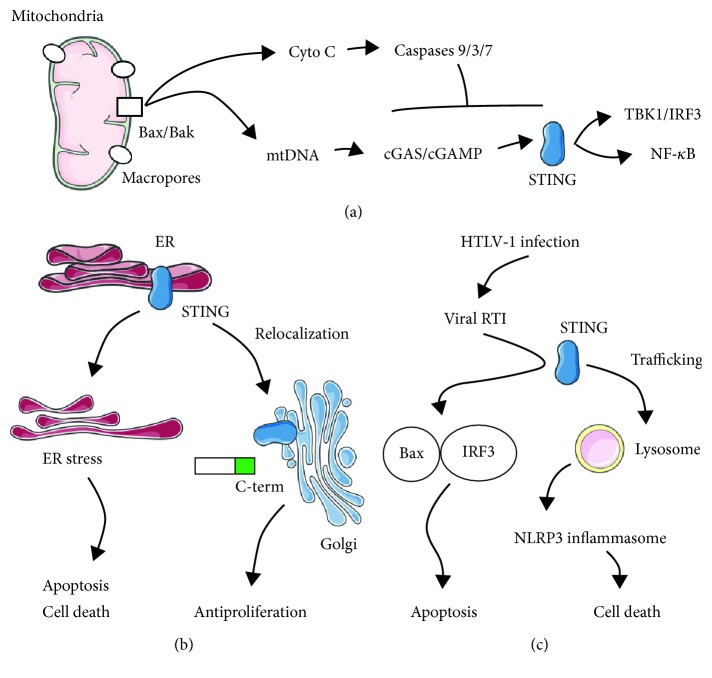
Graphic summary of STING-mediated apoptosis and cell death. (a) Prodeath protein Bak/Bax controls macropore formation in the mitochondrial outer membrane that allows mtDNA efflux into the cytoplasm where it activates cGAS/cGAMP and STING signaling, resulting in inflammatory events via TBK1/IRF3 and NF-*κ*B pathways. Simultaneously, mitochondrial membrane permeabilization by Bax/Bak permits cytochrome C escape that initiates caspases, which is capable of inhibiting STING-mediated apoptosis. (b) STING activation in T lymphocytes could promote ER stress and subsequent apoptosis and cell death. In addition, STING could elicit antiproliferation effects depending on a C-terminal subdomain and requiring relocalization from ER to Golgi. (c) Viral reverse transcription intermediates (RTI) of human T cell leukemia virus type 1 (HTLV-1) could cooperate with STING to promote the IRF3-Bax complex for subsequent apoptosis. STING recognizes cytosolic DNA and traffics to the lysosome, where it activates NLRP3-associated inflammasomes and leads to cell death.

In conclusion, recent discoveries have clearly demonstrated dual outcomes of STING signaling (Figure 1). Besides canonical immune responses, STING signaling can also induce cell death events. The double-faced characteristics of STING requires further exploration and precious regulation before tailoring clinical strategies for associated diseases.
